# Supplementary material for: A whole-animal phenotypic drug screen identifies suppressors of atherogenic lipoproteins
Source: eLife. 2026 Aug 27;14:RP105314. doi: 10.7554/eLife.105314 (PMC13521566; doi:10.7554/eLife.105314)
Supplement: Supplementary file 2 [file elife-105314-code1.zip › JHDL_validation_result/CMP_KSK20240226_01.pdf]

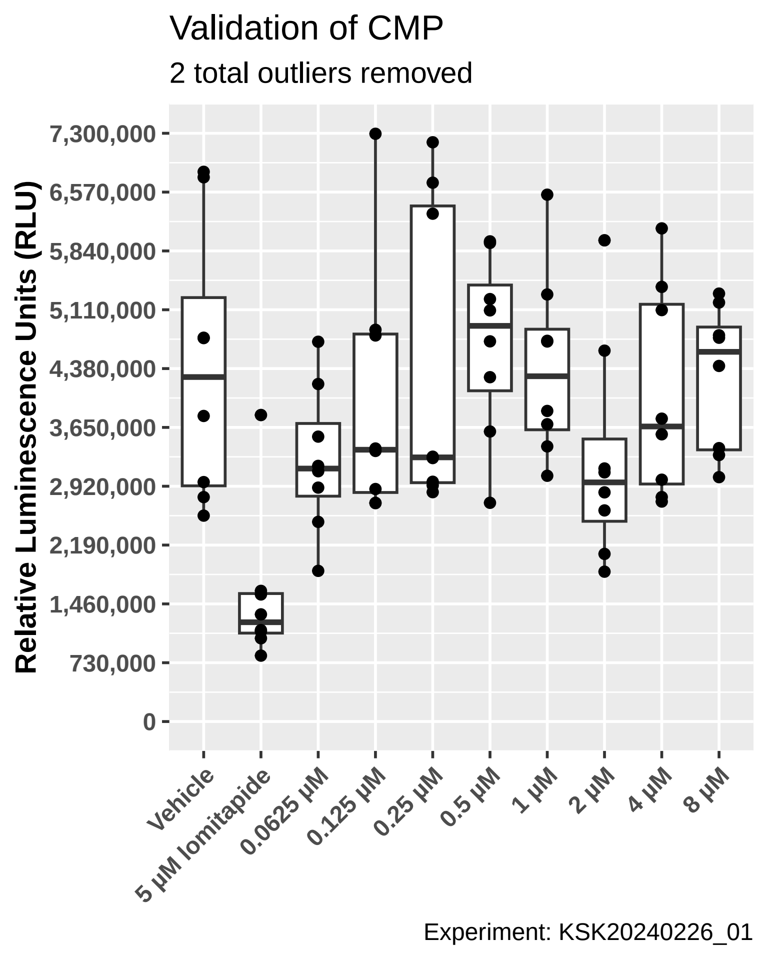
Validation of CMP
2 total outliers removed
Relative Luminescence Units (RLU)
7,300,000
6,570,000
5,840,000
5,110,000
4,380,000
3,650,000
2,920,000
2,190,000
1,460,000
730,000
0
Vehicle
5 µM lomitapide
0.0625 µM
0.125 µM
0.25 µM
0.5 µM
1 µM
2 µM
4 µM
8 µM
Experiment: KSK20240226_01
